# Reconfigurable Selector‐Only Memory (SOM) for Scalable Neuromorphic Computing

**DOI:** 10.1002/advs.75989

**Published:** 2026-06-04

**Authors:** Jin‐Yu Wen, Chuan‐Qi Yi, Ya‐Ru Zhang, Bin‐Hao Wang, Zi‐Xuan Liu, Chun‐Yu Zhou, Hao Tong, Xiang‐Shui Miao

**Affiliations:** ^1^ School of Integrated Circuits Huazhong University of Science and Technology Wuhan China; ^2^ Institute of Artificial Intelligence Huazhong University of Science and Technology Wuhan China; ^3^ Hubei Yangtze Memory Laboratories Wuhan China

**Keywords:** (selector‐only memory, growing‐when‐required (GWR) continual learning), leaky integrate‐and‐fire (LIF) neuron, ovonic threshold voltage, reconfigurable neuromorphic computing

## Abstract

Highly scalable reconfigurable neuromorphic devices are critical for addressing continual‐learning challenges in artificial intelligence. However, the scalability of existing reconfigurable devices is severely constrained by limited operating margins and insufficient process maturity. Here, we propose selector‐only memory (SOM) as a scalable device candidate. Its volatile threshold switching and programmable nonvolatile threshold window are operationally decoupled, and it is compatible with in‐line fabrication and 3D stacking. We demonstrate an In‐doped GeSe SOM that enables neuron–synapse reconfigurability within a single cell. By leveraging intrinsic parasitic capacitance, we implement a capacitor‐free leaky integrate‐and‐fire neuron and validate all‐or‐none firing, integrate‐and‐fire dynamics, and input‐controlled firing‐rate modulation using experiments and an equivalent model. For synapses, we propose a one‐shot subthreshold‐conductance readout method. With a unified reverse‐subthreshold pulse scheme, 16 programmed conductance states are obtained through one‐shot subthreshold readout, and most states remain distinguishable over 10^4^ s. Finally, SOM‐parameter‐based simulations on a Growing‐When‐Required MNIST task achieve 2.67× higher accuracy with 70% of the nodes and shrink to 58% after rollback. These results indicate that SOM provides a promising selector‐derived device concept for scalable reconfigurable neuromorphic hardware.

## Abstract 2

Concept of SOM‐enabled reconfigurable neuromorphic hardware for continual learning and dynamic networks. (a) Schematic of GWR learning with adaptive node growth/shrinkage under evolving classes. (b) 3D‐stackable SOM array concept for scalable integration. (c) Cross‐sectional TEM image of an In‐doped GeSe SOM cell (TE/GeSeIn/BE). (d) Reconfigurable operation principle: neuron‐like spiking dynamics and synapse‐like multilevel states enabled within the same SOM device.

## 1 Introduction

**FIGURE 1:**
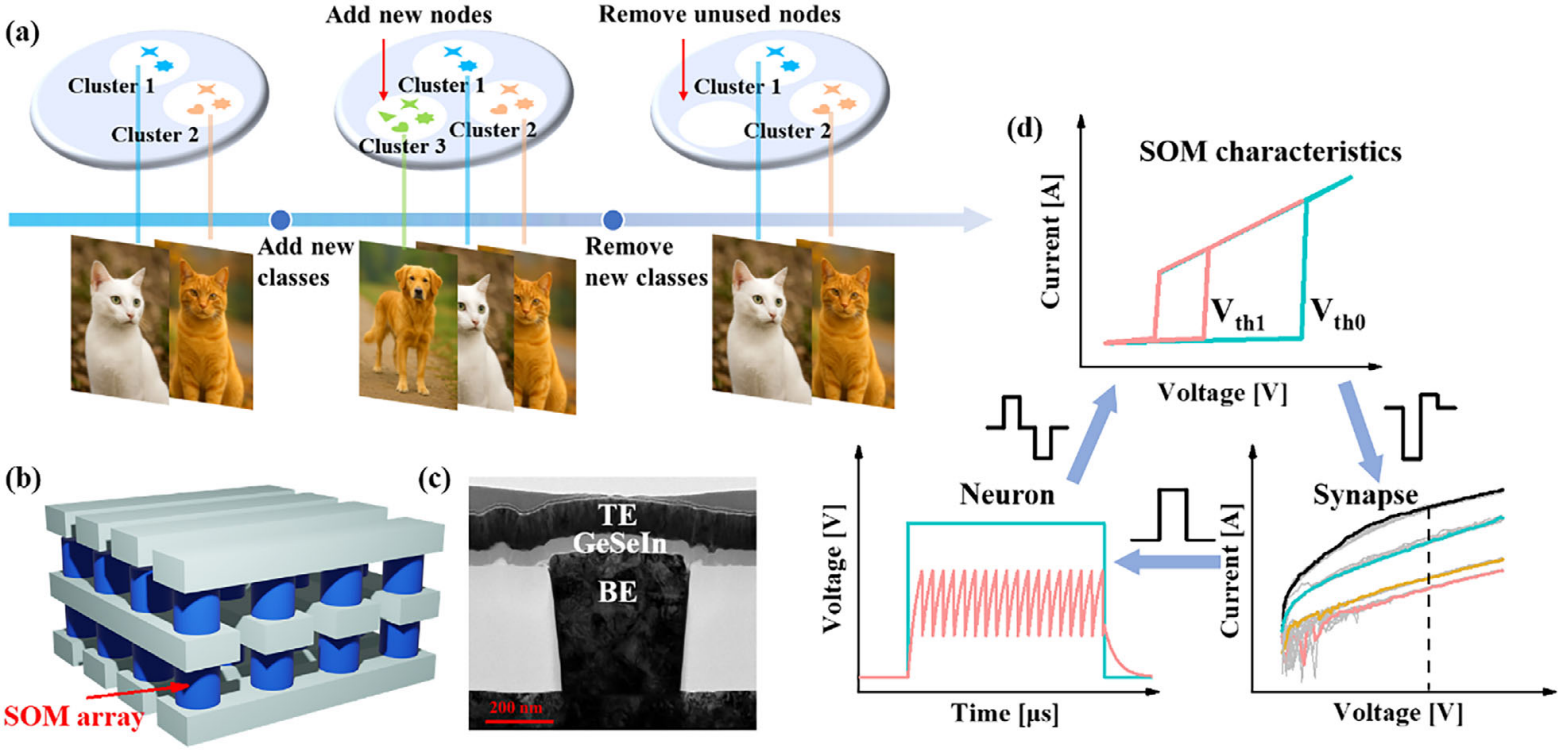
Concept of SOM‐enabled reconfigurable neuromorphic hardware for continual learning and dynamic networks. (a) Schematic of GWR learning with adaptive node growth/shrinkage under evolving classes. (b) 3D‐stackable SOM array concept for scalable integration. (c) Cross‐sectional TEM image of an In‐doped GeSe SOM cell (TE/GeSeIn/BE). (d) Reconfigurable operation principle: neuron‐like spiking dynamics and synapse‐like multilevel states enabled within the same SOM device.

The lack of continual‐learning capability in artificial intelligence (AI) is one of the key bottlenecks limiting its widespread deployment in open environments [1, 2]. To address catastrophic forgetting in conventional neural networks, the Growing‐When‐Required (GWR) network has been proposed in recent years [3, 4]. It adapts network nodes and connection topology as data distributions change (Figure 1a). Reconfigurable‐device‐based neuromorphic hardware can dynamically adjust and allocate network resources [1, 5, 6, 7, 8]. It is therefore viewed as a promising route toward energy‐efficient dynamic networks [6]. Accordingly, reconfigurable devices that can switch between volatile behaviors (for neuron mimicking) and nonvolatile behaviors (for synapse mimicking) are crucial [9, 10, 11], which may enable a transformative paradigm shift toward next‐generation energy‐efficient neural network.

Many reported reconfigurable devices rely on ion‐migration mechanisms, such as electrochemical metallization (ECM) [12, 13, 14, 15] and valence‐change mechanisms (VCM) [9]. Other routes have also been explored, including electrochemical doping [1, 16], ferroelectrics [17], phase‐change materials [18], and spintronics [19]. However, filamentary ion‐migration devices typically require tight control of programming pulses and current compliance to induce spontaneous or non‐spontaneous rupture of the conducting filament [7]. Such an operation makes the devices more susceptible to variations and operational perturbations. As a result, such devices still face challenges in variability and robustness, which limit scalability in large arrays [20] and, consequently, constrain the representational resources available to dynamic networks, undermining their system‐level benefits.

Selector‐only memory (SOM) offers a promising pathway toward highly scalable dynamic networks. SOM is built on ovonic threshold switching (OTS) devices [21], which enable a programmable nonvolatile threshold window through bipolar write/erase operations [22, 23, 24]. Under unipolar operation, SOM behaves as a volatile switch with an approximately fixed threshold [25]. When the reverse operation is introduced, the switching remains volatile, while a nonvolatile threshold window is established and retained until the next conduction event [26]. Hence, in terms of operation, the volatile switching and the nonvolatile threshold window are relatively decoupled. This feature reduces the risk of mode confusion caused by device variation or pulse fluctuations. Moreover, SOM has been developed as an in‐line selector element and has been demonstrated at high density [27, 28, 29] (Figure 1b), such as 256 Gb capacity [30] and 14 nm feature size [31]. This indicates clear advantages in scalability and process feasibility for reconfigurable arrays. In addition, the low leakage current of SOM is beneficial for lowering static power and approaching ultra‐low‐power neuromorphic operation [32]. Nevertheless, SOM‐based studies that explicitly demonstrate neuron–synapse reconfigurability remain limited.

In this work, we employ an In‐doped GeSe SOM device to realize reconfigurable neuron and synapse functions and validate its potential for continual learning in dynamic networks. First, with 3% In incorporation into GeSe, we obtain SOM devices with an advantageous threshold window, good threshold uniformity, and low leakage current. Next, a capacitor‐free leaky integrate‐and‐fire (LIF) neuron is implemented using the SOM parasitic capacitance, and its firing dynamics and rate modulation are validated experimentally and by an equivalent model. Besides, for multilevel synaptic weight representation, we propose a subthreshold‐conductance readout strategy. We combine it with reverse subthreshold pulses to achieve multilevel tuning and evaluate the retention of programmed conductance states within a single SOM device. Finally, using SOM‐parameter‐based simulations on a GWR dynamic handwritten‐digit recognition task, we demonstrate system‐level mitigation of catastrophic forgetting with low structural overhead. Collectively, this device–circuit–algorithm co‐verification highlights SOM as a highly scalable building block for dynamic neuromorphic hardware targeting edge continual‐learning applications.

## 2 Results and Discussion

### 2.1 Principle of Switching SOM Between Neuron and Synapse Modes

As shown in Figure 1c, the fabricated SOM device adopts a vertical sandwich structure of electrode/chalcogenide/electrode. The chalcogenide layer is a 3% In‐doped GeSe film. The active‐layer composition and layer‐stack integrity are further verified by cross‐sectional TEM and EDS analysis (Figure S1). Compared with Te‐ or S‐based chalcogenide systems, the GeSe system in this work exhibits a wider and more stable threshold‐voltage window [26], providing a device basis for switching between volatile threshold switching and nonvolatile threshold memory modes within the same device. In addition, moderate In doping markedly improves write/erase cycling endurance [33]: our In‐doped GeSe SOM devices operate stably beyond 10^5^ write/erase cycles under identical operating conditions (Figure S2), ensuring reliability for repeated reconfiguration operations.

Based on this structure, the reconfigurability of SOM is illustrated in Figure 1d. First, when SOM is programmed only with same‐polarity positive pulses, the device exhibits a stable low‐threshold threshold‐switching behavior: it remains in a high‐resistance state below the threshold voltage, rapidly transitions to a low‐resistance state once the threshold is crossed, and spontaneously returns to the high‐resistance state after the bias is removed. When this volatile threshold switching is combined with charge/discharge dynamics of a parallel capacitance, the high‐/low‐resistance phases naturally correspond to membrane‐potential integration and rapid discharge in a LIF neuron. Notably, owing to the sandwich geometry, SOM intrinsically possesses an effective parasitic capacitance in parallel with the switching element. Therefore, a compact capacitor‐free LIF neuron circuit can be implemented using only an external series resistor.

When bipolar write/erase pulses are applied to the same device, SOM can be reconfigured into a memory cell with a nonvolatile threshold window. A negative reset pulse drives defects toward a more localized configuration [34, 35, 36] (i.e., a recovery‐like process), shifting the threshold toward a higher forming voltage regime. During the subsequent positive read, the threshold voltage increases markedly from the low‐threshold state (V_th1_) to the high‐threshold state (V_th0_). Importantly, when the device remains in the high‐threshold state without further switching events, the threshold state is nonvolatile, enabling nonvolatile threshold‐state switching. Furthermore, by superimposing a small reverse subthreshold pulse during the reset process, one can selectively tune the fraction of “fast” and “slow” defects [37, 38], thereby modulating the local structural amorphization degree and enabling multilevel nonvolatile resistance states. Under this framework, unipolar operation corresponds to the volatile threshold‐switching regime widely reported for OTS devices [39, 40, 41], whereas bipolar operation establishes the programmable threshold‐window effect reported for SOM [21, 28, 34]. Because SOM is based on amorphous chalcogenide materials, the microscopic origin of the threshold‐window effect remains under active discussion in the literature. Therefore, the present work adopts a literature‐consistent interpretation of polarity‐dependent local defect/structural evolution to explain the observed behavior, while focusing primarily on functional reconfigurability rather than a definitive mechanistic determination. Consequently, a single SOM device can be configured—via bias sequences—into multiple functional modes including a volatile neuron, a nonvolatile synapse, and a multilevel synapse.

### 2.2 Basic Electrical Characteristics of In‐Doped GeSe SOM

**FIGURE 2:**
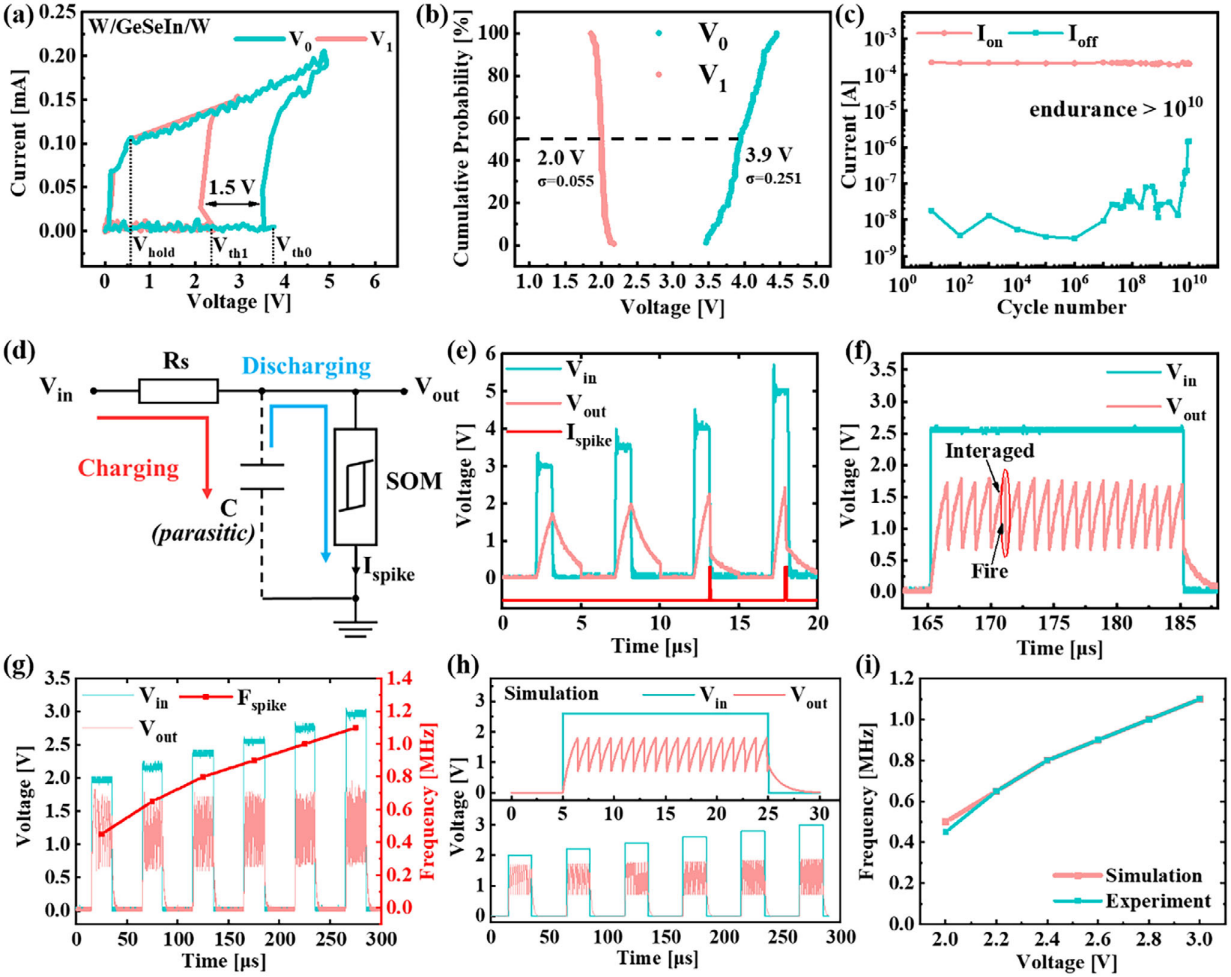
Electrical characteristics of the In‐doped GeSe SOM and capacitor‐free LIF neuron operation enabled by intrinsic parasitic capacitance. (a) Dual‐threshold I–V characteristics showing a large threshold window. (b) Threshold‐voltage distributions over cycling. (c) Switching endurance. (d) Capacitor‐free LIF neuron circuit schematic. (e,f) All‐or‐none firing and leaky integrated‐and‐fire. (g) Input‐amplitude‐controlled firing‐rate modulation. (h,i) Equivalent‐model simulation and experiment–simulation agreement for the firing rate versus input voltage.

We first systematically characterize the volatile and nonvolatile threshold characteristics of SOM devices. Figure 2a shows the representative current–voltage (I–V) characteristics: after a positive set operation, the device resides in a low‐threshold state with threshold voltage V_th1_; after a negative reset pulse, it is reprogrammed into a high‐threshold state with threshold voltage V_th0_. In our In‐doped GeSe SOM devices, the threshold window between V_th0_ and V_th1_ is 1.5 V. A larger threshold window is beneficial for multilevel nonvolatile states and improves state separability under noise and process variations. Figure 2b summarizes threshold‐voltage statistics over 100 consecutive cycles under identical write/erase pulses, where the low‐threshold state shows particularly high uniformity (σ = 0.055)—an essential prerequisite for stable LIF neuron operation. Furthermore, device currents measured at 3 V (on‐state read) and 1 V (off‐state read) are tracked as a function of cycling number (Figure 2c), showing a stable on/off ratio up to ∼10^10^ switching cycles without evident degradation, indicating excellent endurance for reliably emulating massive spiking events. In addition, as shown in Figure S3, the turn‐on and turn‐off times reach 2.8 and 6 ns, respectively, highlighting the ultrafast response of SOM devices.

### 2.3 Capacitor‐Free LIF Neuron: All‐or‐None Response, Integration, and Frequency Modulation

Building on these device characteristics, we construct a capacitor‐free LIF neuron circuit based on SOM (Figure 2d). The intrinsic parasitic capacitance of the SOM (∼100 pF) serves as the membrane capacitance, and a series resistor with a value of 10 kΩ is used, forming a typical RC–threshold‐switching (RC–TS) configuration. The input voltage V_in_ and output voltage V_out_ are monitored by an oscilloscope. Under different stimuli, the circuit exhibits hallmark neuronal behaviors including all‐or‐none response, integrate‐and‐fire dynamics, and firing‐rate modulation.

Figure 2e demonstrates the all‐or‐none firing behavior. With 1 µs input pulses, when the input amplitude is 3 or 3.5 V, the node voltage does not cross the threshold and the neuron remains quiescent without spikes; in the absence of sustained stimulation, the membrane potential gradually leaks back to the resting state. When the input amplitude increases to 4 and 5 V, the membrane potential crosses the threshold and triggers spiking, and stronger stimuli lead to a shorter firing latency—mimicking selective neuronal responses to different stimulus intensities. Figure 2f shows integrate‐and‐fire behavior: when the neuron receives multiple stimuli within a short time window, each stimulus contributes an incremental integration, eventually driving the membrane potential above threshold to generate a spike. Subsequently, the membrane potential rapidly releases from threshold and decays below the hold voltage; during this interval, the neuron remains in a refractory state and cannot be re‐excited, analogous to the biological refractory period. Figure 2g further indicates that the firing rate can be tuned by the input amplitude: under sustained stimulation similar to Figure 2f, a higher input voltage accelerates charging and substantially increases firing frequency, reflecting a canonical rate‐coding mechanism.

To quantitatively capture SOM‐neuron dynamics, we develop a numerical model using experimentally measured circuit parameters. In the simulation, the charging process is governed by the series resistance and parasitic capacitance, while the discharge process is fitted using the experimentally measured average time constant extracted from 20 discharge events. By tuning the model parameters, we reproduce the experimental firing rate at an input voltage of 2.6 V (upper panel of Figure 2h) and further simulate firing‐rate variations for input amplitudes ranging from 2.0 to 3.0 V (lower panel of Figure 2h). Figure 2i compares experimental and simulated firing‐rate–voltage curves, showing close agreement in both trend and absolute values, indicating that the equivalent model reliably captures key SOM‐neuron dynamics and supports subsequent array‐level system simulations and evaluations.

### 2.4 Multilevel Synapse via Subthreshold‐Conductance Readout: One‐Shot Read, Separable States, and Low Energy

Although the nonvolatile threshold window of SOM can encode memory states, directly using “conducting vs. non‐conducting” as the state indicator becomes inefficient for multilevel synaptic weights. Conventional threshold‐based readout requires applying read pulses with amplitudes between the high‐ and low‐threshold states and counting switching (conduction) events to determine the threshold state. To discriminate n states, at least n−1 reads are typically required, and repeated switching introduces time overhead, unnecessary energy consumption, and potentially reduced device reliability. To address this limitation, we propose a subthreshold‐conduction readout scheme that maps distinct threshold states to directly readable subthreshold resistance states, thereby significantly reducing read energy while maintaining state stability.

**FIGURE 3:**
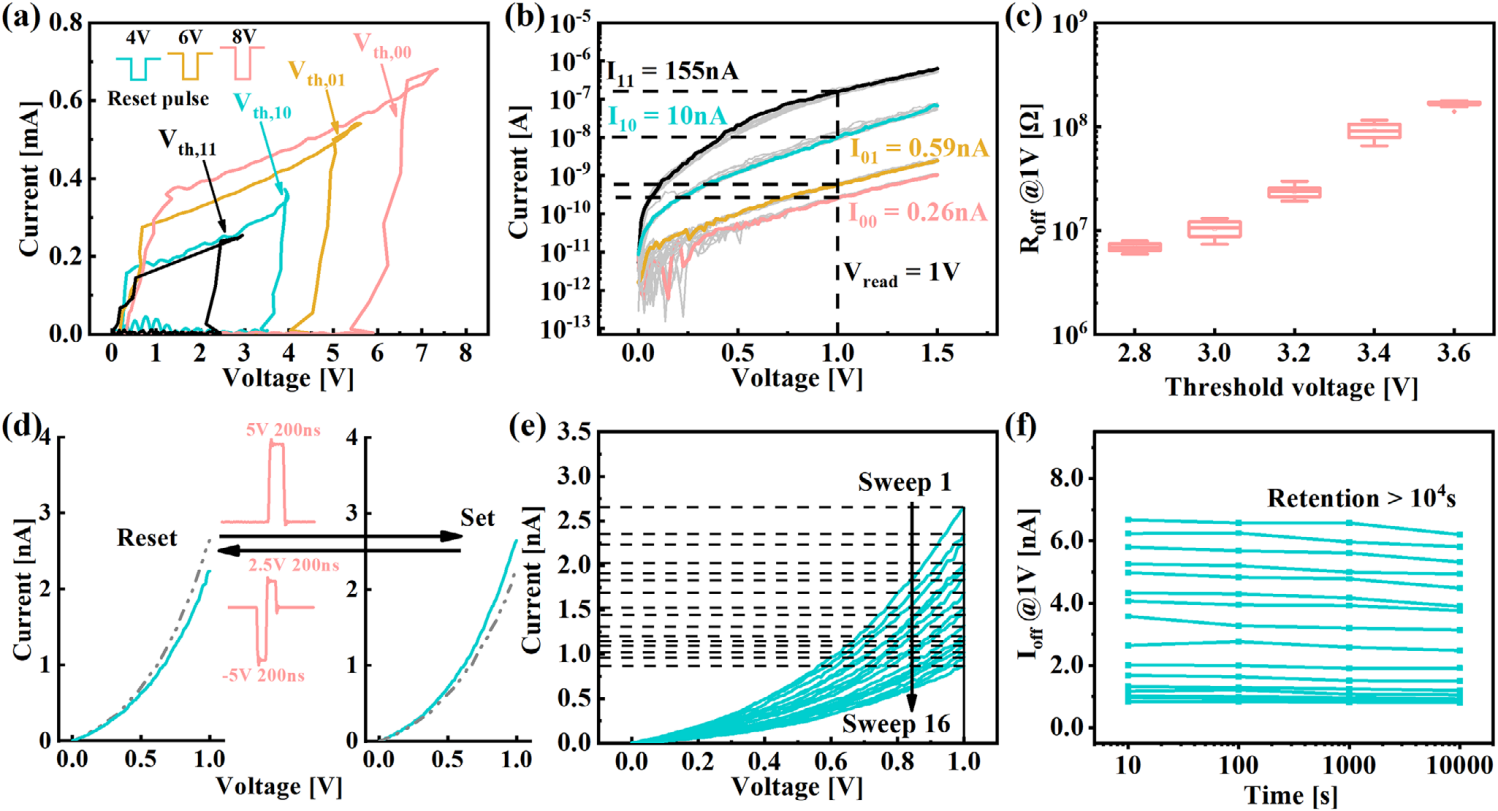
Multilevel synapse implementation using SOM via subthreshold‐conductance readout and unified pulse programming. (a) Multiple threshold states programmed by reset pulses. (b) Subthreshold I–V curves enabling one‐shot state discrimination at a fixed read voltage. (c) Correlation between threshold state and extracted high‐resistance level. (d,e) Pulse scheme and resulting multilevel conductance states. (f) Retention of 16 programmed conductance states.

Figure 3a shows that at least four stable threshold states can be achieved by using reset pulses with different amplitudes; a larger reset amplitude produces a higher threshold voltage. Figure 3b presents the subthreshold I–V characteristics of these threshold states, where a higher threshold voltage corresponds to a smaller subthreshold current (i.e., a higher resistance) at a given read voltage. This verifies that threshold information can be transduced into separable subthreshold high‐resistance states, enabling state discrimination via a single current read. To evaluate readout reliability, we repeat I–V scans 10 times for each state. When the read voltage is below 0.5 V, the current approaches the noise floor and noticeable dispersion appear within the same state; when the read voltage is set to 1 V, the cycle‐to‐cycle consistency improves significantly. Figure 3c further correlates the subthreshold conductance measured at 1 V with threshold voltage, showing a clear monotonic relationship and small intra‐state variation, confirming robust multistate discrimination under the proposed readout scheme.

We further demonstrate multilevel tuning of subthreshold conductance using identical reverse subthreshold pulses. Prior studies have shown that varying the amplitude of reverse subthreshold pulses can tune the ratio of fast/slow defects in OTS materials [42], thereby modulating the amorphization degree and enabling multilevel conductance states. Following this rationale, we confirm that under the high‐threshold state, repeatedly applying the same pulse sequence can similarly modulate the amorphization degree in SOM. Specifically, a negative reset pulse of −5 V with a 200 ns width is applied together with a reverse tuning pulse of 2.5 V; by repeating this unified pulse sequence and reading subthreshold conductance at 1 V, 16 programmed conductance states are obtained (Figures 3d,e), demonstrating that multilevel tuning can be achieved with a uniform driving scheme suitable for arrays. Benefiting from the ns‐scale switching and nS‐scale conductance, the synaptic inference energy is estimated to reach the sub‐fJ regime. Based on an evaluation using a 1 V read voltage, a 50 ns read pulse width, and a 10 nS conductance, the proposed SOM synapse achieves a sub‐fJ energy consumption per inference. Finally, retention of all 16 programmed conductance states is evaluated under the same 1 V subthreshold read condition (Figure 3f). Most programmed states remain distinguishable over 10^4^ s, verifying the feasibility of nonvolatile multilevel‐state representation in the present SOM device. However, reduced read margins and partial state overlap are observed in the low‐conductance region, indicating that fully reliable 16‐level retention has not yet been achieved. These data therefore support SOM‐based multilevel‐state feasibility within the measured window, while further optimization of low‐conductance‐state separation, elevated‐temperature retention, and retention after repeated multilevel cycling is still required.

From the viewpoint of a reconfigurable neuromorphic device, SOM provides a novel device solution for reconfigurable neuromorphic hardware. Its main strengths arise from the compact selector‐derived two‐terminal structure, fabrication relevance, and programmable threshold states. Beyond synaptic‐state encoding, the threshold programmability of SOM can be naturally mapped to neuron excitability and node‐level gating, which may support richer neuron functions and dynamic‐network operations such as adaptive node growth or reduction. Nevertheless, the present SOM implementation still faces clear challenges in available state count, multilevel program endurance, and longer‐term retention, which should be further optimized before practical neuromorphic deployment.

### 2.5 Dynamic GWR Continual‐Learning Simulations: Accuracy–Resource Advantage Enabled by Adaptive Node Growth/Shrinkage

**FIGURE 4:**
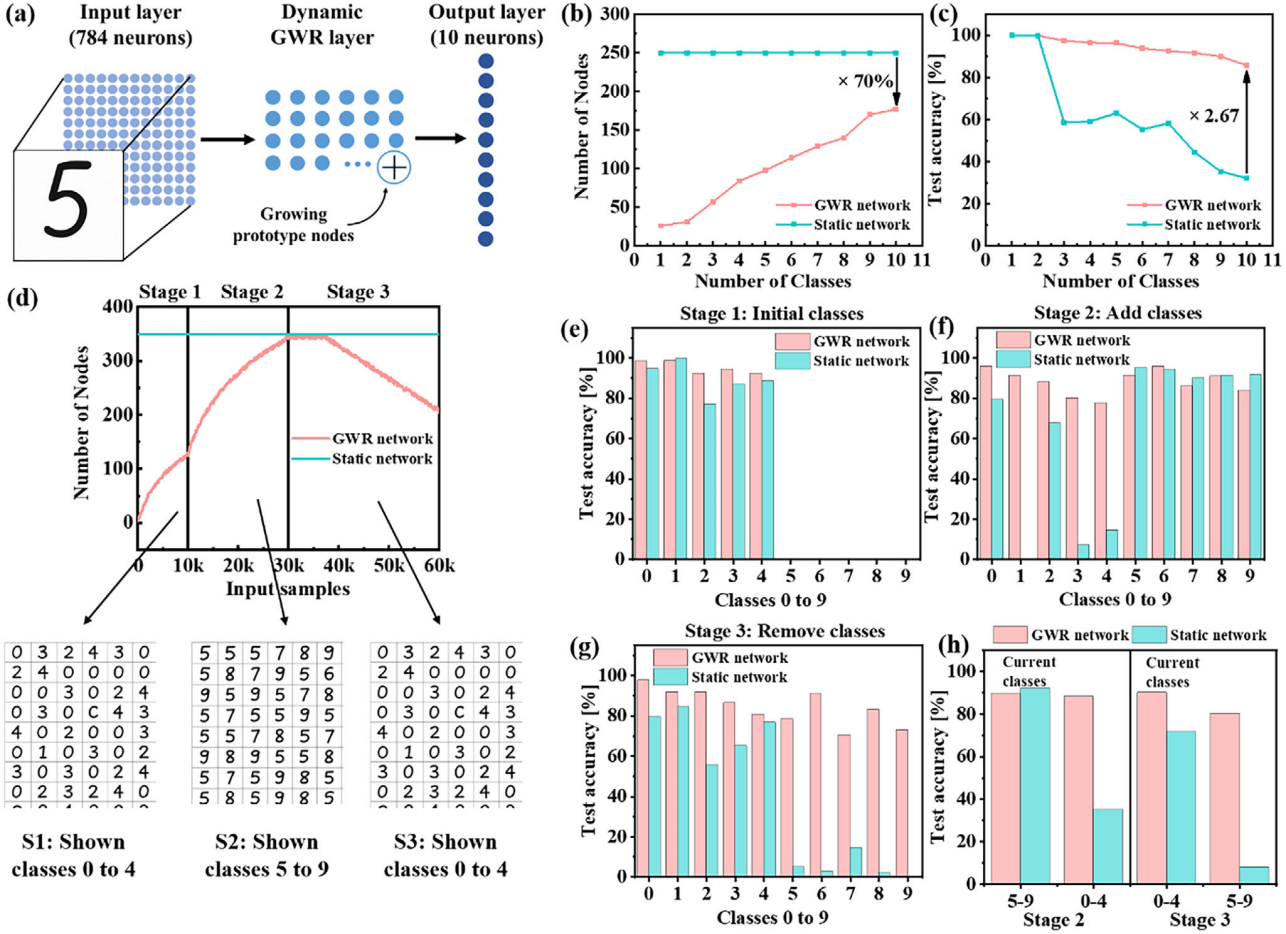
System‐level validation by SOM‐parameter‐based simulations on a GWR dynamic handwritten‐digit recognition task (MNIST). (a) Network schematic with a dynamic GWR layer. (b,c) Node count and accuracy during class‐incremental learning (dynamic vs. static). (d) Abrupt class‐shift task with growth and shrinkage. (e–g) Per‐class accuracy after each stage. (h) Summary showing improved accuracy with reduced structural overhead in the dynamic network.

Implementing both neuron and synapse functions within a single device enables on‐demand reconfiguration of network representational capacity and topology as tasks evolve, providing greater structural flexibility for dynamic neural networks. The Growing‐When‐Required (GWR) network is a representative framework: based on competitive Hebbian learning (competitive Hebbian learning), it dynamically creates nodes and their interconnections during unsupervised learning and can remove redundant nodes when necessary, thereby approximating the input space distribution more finely. To validate its effectiveness in continual‐learning scenarios, we construct a GWR dynamic network and benchmark it against a static self‐organizing network that uses the same competitive Hebbian update rule; the baseline network has a fixed number of nodes and is randomly initialized before training. We evaluate both networks on the MNIST dataset, as summarized in Figure 4a.

To systematically assess continual‐learning capability, we design two simulation tasks using neuron/synapse parameters extracted from SOM experiments. The first task is class‐incremental learning: classes 0–9 are introduced sequentially in stages, starting from stage 1 containing only class 0, and adding one new class per stage; the number of training samples per stage is fixed at 6000. During training, the GWR network grows its node count on demand according to the complexity of the input distribution, while the static baseline uses a fixed node count of 250. After each stage, we quantify (i) the network node count and (ii) the classification accuracy on the set of previously seen classes (Figure 4b,c). Figure 4b shows adaptive node growth in GWR, whereas the static baseline remains fixed at 250 nodes. Figure 4c demonstrates that, in the expanding‐class MNIST task, GWR achieves a 2.67× higher final accuracy than the static baseline while using only 70% of the nodes. These results indicate that on‐demand structural expansion allocates additional representational capacity for new classes, effectively alleviating catastrophic forgetting while reducing structural redundancy.

The second task evaluates structural adaptability under an abrupt class shift: the network is first trained on half of the classes; after convergence, the remaining classes are introduced for continued training; once the network stabilizes, the latter half of the classes is removed so that the task distribution returns to the initial class set (Figure 4d). This process explicitly tests node growth and shrinkage as well as performance retention under both class increase and class decrease. Accuracy comparisons across stages are shown in Figure 4e–h. In stage 1 (Figure 4e), the static and GWR networks exhibit comparable accuracy; after new classes are added in stage 2 (Figure 4f), GWR maintains recognition of both previously learned and newly introduced classes, whereas the static baseline suffers pronounced performance degradation on learned classes, indicating catastrophic forgetting. When classes roll back in stage 3 (Figure 4g), GWR recovers faster on the initial class set. The final summary (Figure 4h) shows that GWR achieves higher overall accuracy while using only 58% of the nodes after shrinkage. Overall, Figure 4e–h demonstrate that GWR consistently offers a superior accuracy–structural‐cost trade‐off throughout abrupt class expansion and contraction. These results suggest that dynamic networks supported by SOM reconfigurable units can adapt representational capacity to task demands, providing an initial system‐level indication of the potential value of SOM‐enabled dynamic neuromorphic hardware.

Finally, the influence of low‐frequency noise (LFN) and random telegraph noise (RTN) should also be considered in the device‐to‐system mapping. For SOM synapses, temporal fluctuations in the subthreshold read current may introduce weight‐state uncertainty; similar RTN behavior has been reported in Ge_x_Se_1__−__x_ OTS selectors [43]. For SOM neurons, threshold fluctuation can lead to spike‐time jitter and firing‐rate dispersion, and related LFN/RTN‐induced instability has also been discussed in ferroelectric memory and neuromorphic devices [44]. In the GWR network, these variations may further affect winner selection, node activation, node growth/shrinkage decisions, and class‐boundary stability [45]. Therefore, future SOM‐based simulations should incorporate noise‐induced read and firing variations, while possible mitigation strategies include material/process optimization, larger read margins, fewer effective level when necessary, redundant coding, temporal averaging, calibration, and noise‐aware network design [46].

## 3 Conclusions

This work proposes a single‐device neuron–synapse reconfigurable neuromorphic scheme based on selector‐only memory (SOM) and further validates its advantages for continual‐learning dynamic‐network tasks. At the device level, 3% In‐doped GeSe SOM provides a nonvolatile threshold window of 1.5 V and ultrafast threshold switching with 2.8 ns turn‐on and 6 ns turn‐off, together with high threshold uniformity (σ = 0.055) and high switching endurance (10^10^), supporting reconfigurability and high‐frequency event‐driven operation. At the neuromorphic‐function level, a capacitor‐free LIF neuron is realized with key dynamics including all‐or‐none firing, integrate‐and‐fire dynamics, and firing‐rate modulation, and the equivalent model reproduces the firing‐rate–voltage relationship; a subthreshold‐conductance readout strategy enables one‐shot high‐resistance‐state discrimination, yielding 16 programmed conductance levels under a unified pulse scheme with most states distinguishable over 10^4^ s, and the per‐read energy is estimated to reach the sub‐fJ regime at the device level. At the system level, MNIST‐based GWR simulations show that in class‐incremental learning, the dynamic network achieves 2.67× higher final accuracy using 70% of the nodes of the static baseline; in class expansion–contraction learning, the dynamic network exhibits node growth and shrinkage and maintains superior overall performance while using 58% of the nodes after contraction. Collectively, these results establish SOM as a promising selector‐derived route toward scalable reconfigurable neuromorphic hardware for dynamic‐network applications.

## 4 Experimental Section

### 4.1 Device Fabrication and Electrical Characterization

A T‐shaped SOM device featuring a 450‐nm‐diameter bottom electrode was fabricated with CMOS device fabrication processes, as illustrated in Figure 1c. The OTS layer and top electrode (TE) were subsequently defined by UV photolithography. After pattern development, a 50‐nm GeSeIn film and a 100‐nm W TE were sequentially deposited via magnetron sputtering. The GeSeIn layer was formed by co‐sputtering from GeSe and In targets. Electrical measurements were performed using a Keysight B1500A semiconductor parameter analyzer equipped with a B1530A waveform generator/fast measurement unit (WGFMU). To mitigate device stress under high operating currents, a 10‐kΩ series resistor was introduced. Microstructural and compositional analyses were carried out by transmission electron microscopy (TEM).

## Conflicts of Interest

The authors declare no conflict of interest.

## Supporting information




**Supporting File**: advs75989‐sup‐0001‐SuppMat.docx.

## Data Availability

The data that support the findings of this study are available from the corresponding author upon reasonable request.
