# Supplementary material for: Reconfigurable Selector‐Only Memory (SOM) for Scalable Neuromorphic Computing
Source: Adv Sci (Weinh). 2026 Jun 4;13(48):e75989. doi: 10.1002/advs.75989 (PMC13336487; doi:10.1002/advs.75989)
Supplement: Supplementary file 1 [file ADVS-13-e75989-s001.docx]

Supporting Information

Reconfigurable Selector-Only Memory (SOM) for Scalable Neuromorphic Computing

Jin-Yu Wen, Chuan-Qi Yi, Ya-Ru Zhang, Bin-Hao Wang, Zi-Xuan Liu, Chun-Yu Zhou, Hao Tong* and Xiang-Shui Miao

**Supplementary Fig. S1.** Cross-sectional TEM and EDS characterization of the In-doped GeSe SOM device. (a) Cross-sectional TEM image with EDS elemental maps of Se, Ge, and In, showing the elemental distribution in the active layer. (b) EDS line-scan profiles across the film thickness, confirming a Se/Ge-rich active layer with incorporated In dopant and the layer-stack integrity.

**Supplementary Fig. S2.** Evolution of the threshold voltages during bipolar program/erase cycling of the SOM device. The high-threshold state and low-threshold state are extracted after alternating write/erase operations, demonstrating stable dual-threshold operation and a maintained threshold window under bidirectional cycling.

**Supplementary Fig. S3.** Switching-speed characterization of the SOM device measured under pulsed operation. Left: turn-on (set) transient showing the rapid rise of the output voltage/current response, with the extracted on-switching time T_on_ = 2.8 ns (highlighted window). Right: turn-off (reset) transient showing the fast recovery to the OFF state, with the extracted off-switching time T_off_ = 6 ns (highlighted window). The insets zoom into the rising/falling edges used to determine the switching times.
